# Preserving nanoscale features in polymers during laser induced graphene formation using sequential infiltration synthesis

**DOI:** 10.1038/s41467-020-17259-5

**Published:** 2020-07-20

**Authors:** David S. Bergsman, Bezawit A. Getachew, Christopher B. Cooper, Jeffrey C. Grossman

**Affiliations:** 1 Department of Materials Science and Engineering, Massachusetts Institute of Technology, 77 Massachusetts Ave, Cambridge, MA USA; 2 Department of Chemical Engineering, Stanford University, 443 Via Ortega, Stanford, CA USA

**Keywords:** Graphene, Nanocomposites, Organic-inorganic nanostructures, Water resources

## Abstract

Direct lasing of polymeric membranes to form laser induced graphene (LIG) offers a scalable and potentially cheaper alternative for the fabrication of electrically conductive membranes. However, the high temperatures induced during lasing can deform the substrate polymer, altering existing micro- and nanosized features that are crucial for a membrane’s performance. Here, we demonstrate how sequential infiltration synthesis (SIS) of alumina, a simple solvent-free process, stabilizes polyethersulfone (PES) membranes against deformation above the polymers’ glass transition temperature, enabling the formation of LIG without any changes to the membrane’s underlying pore structure. These membranes are shown to have comparable sheet resistance to carbon-nanotube-composite membranes. They are electrochemically stable and maintain their permeability after lasing, demonstrating their competitive performance as electrically conductive membranes. These results demonstrate the immense versatility of SIS for modifying materials when combined with laser induced graphitization for a variety of applications.

## Abstract (web summary)

High temperatures induced during lasing can deform the substrate polymer used for fabrication of electrically conductive membranes. Here, the authors show that sequential infiltration synthesis of alumina stabilizes polyethersulfone (PES) membranes against deformation above the polymers’ glass transition temperature.

## Introduction

Polymeric membranes play a critical role in a range of technologies, including water filtration, by providing a low-cost method of separating one or more species from their surrounding solvent^1^. To maximize membrane performance, the surface properties of the membranes must be precisely controlled^2^. One strategy to enhance the properties of polymeric membranes is to make them electrically conductive, which provides an additional mechanism for combating surface fouling^3–7^, inactivating viruses and bacteria^8,9^, and electrochemically removing heavy metal contaminants^10^. Several approaches have been explored for the production of conductive membranes, including the use of conductive polymers like polyaniline and polypyrrole^11^ and the deposition of carbon nanotubes (CNT) on the surface of polymeric membranes via vacuum filtration^3,5,8,9^. Membranes made out of deposited CNT have been further improved by crosslinking using polyvinyl alcohol (PVA)^6,10^ or polyaniline^12^ to increase their stability. However, conductive polymers like polyaniline and polypyrrole suffer from low conductivity and the vacuum filtration step required for CNT membrane fabrication limits the scalability of that approach. Therefore, there is a critical gap in our ability to produce conductive membranes or, more broadly, any conductive nanostructured materials using scalable, roll-to-roll compatible methods.

Direct lasing of polymeric membranes to form laser-induced graphene (LIG)^13^ offers a scalable and potentially cheaper alternative for the fabrication of conductive membranes. LIG is a porous graphitic film formed through the thermochemical conversion of polymeric materials upon exposure to a CO_2_ infrared laser. Because this process is rapid, can be performed in ambient conditions, is compatible with roll-to-roll processing, can be created from a wide variety of polymers^14^, and produces a porous graphene structure with high conductivity, LIG has been explored for various applications, including energy storage^15,16^, electrocatalysis^17,18^, sensing^17,19^, and the production of antifouling devices^17,20,21^. The antifouling properties of LIG, in particular, have been demonstrated on polymer membrane substrates^22^. Despite its versatility, however, this lasing process can result in the deformation of the original substrate material, either in the form of exfoliation of the lased regions or the softening of the subsurface polymer that is not pyrolyzed. These structural changes distort any existing polymer structure and limit its use on thin and porous polymer materials, such as filtration membranes, where maintaining an underlying structure is critical to performance. To exploit this scalable process to its fullest potential, there is a need for techniques that allow lased materials to maintain their nano and micro-scale features and structure during laser scribing. More broadly, there is a significant gap in approaches that allow nano and micro-scale features in polymers to be maintained during processes that exceed the glass transition temperature of the polymer.

Modification of the polymer substrate could prevent structural loss during lasing while simultaneously introducing an additional lever for control over materials’ properties. Sequential infiltration synthesis (SIS) is one such modification strategy that could be adapted to a scalable roll-to-roll process^23^. Similar to atomic layer deposition (ALD), SIS involves the sequential exposure of a material to alternating vapors of a reactive organometallic precursor and a counter reactant^24,25^. While ALD is performed on a non-porous substrate to grow inorganic films^26^, SIS is performed on polymers, where the diffusion of reactants into the polymer can change its chemical and mechanical properties, increasing its tensile strength^27^, changing its permeability to certain molecules^28^, improving its conductivity^29^, or allowing it to maintain its structure during annealing^30–33^.

ALD, and similar techniques like SIS, have been widely and safely used for decades, both in industry and academic research^34^. Recent work has also highlighted the potential for these approaches to be performed using roll-to-roll atmospheric pressure systems^23,35^. Thus, the combination of SIS and laser pyrolysis is a potentially scalable drop-in technology for converting existing polymer membranes into conductive membranes.

**Fig. 1:**
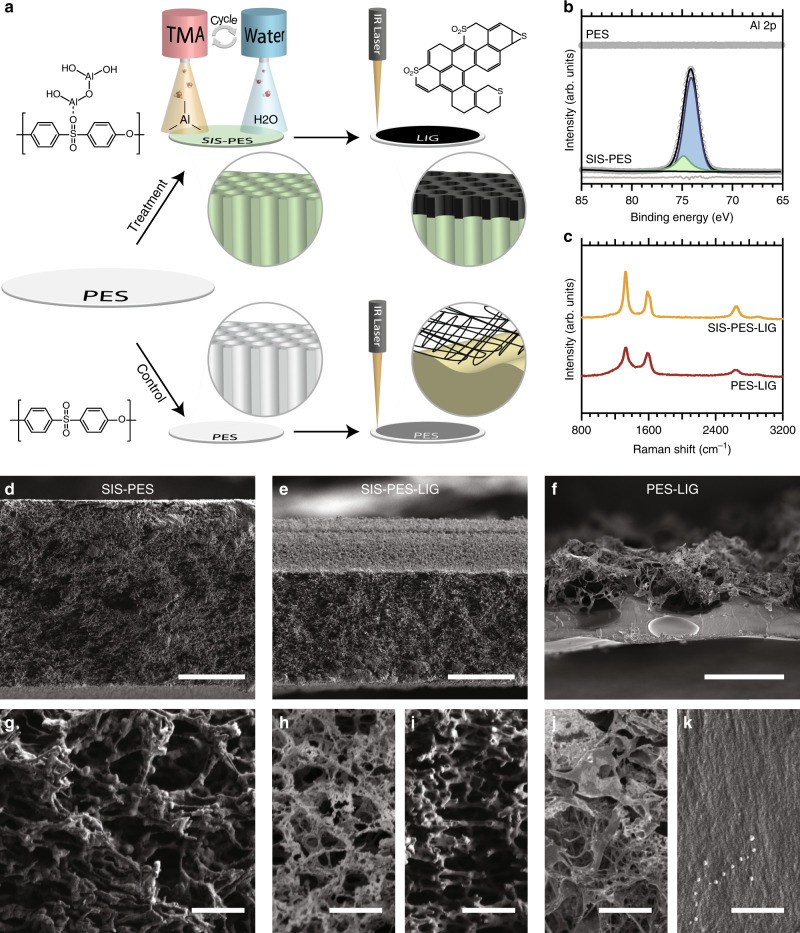
The impact of sequential infiltration synthesis on polymer structure during laser pyrolysis. **a** Schematic illustration of sequential infiltration synthesis of alumina into PES membranes using TMA and water, followed by laser-induced graphitization of the membrane with an IR laser (top). Laser-induced graphitization of control membranes is shown in the bottom half. **b** XPS fine scans of bare PES membranes and SIS-treated membranes, where an Al 2p peak is present only after SIS treatment. **c** Raman spectra of lased membranes with and without SIS treatment. **d** Cross-section SEM image of SIS-treated PES membrane prior to lasing (scale bar 50 µm) and **e** after lasing (scale bar 50 µm). **f** Cross-section SEM image of untreated PES membrane after lasing (scale bar 50 µm). **g**–**k** Higher magnification SEM images of the membrane cross-sections (scale bars 2 µm, 2 µm, 2 µm, 4 µm, 2 µm). **h** The top, brighter portion, and **i** the bottom, darker portion of the SIS-PES-LIG membrane. **j** The top and **k** bottom portions of the PES-LIG membrane.

In this work, we show how an SIS process can allow for the creation of conductive and electrochemically stable LIG coatings on porous polymer substrates without loss of the underlying porosity, using polyethersulfone (PES) microfiltration membranes as a prototypical example. First, the PES membranes are treated using the SIS process with alternating pulses of trimethylaluminum (TMA) and water (Fig. 1a), which are allowed to soak into the polymer membranes, generating alumina and converting the polymers into an organic–inorganic hybrid material. An alumina process was chosen because it is a prototypical example for both ALD^36^ and SIS^37^. The SIS-treated PES membranes are then irradiated with a CO_2_ laser to induce photothermal transformation of the top surface to LIG (Fig. 1a). We show that this process allows for the successful conversion of PES into LIG without loss of the underlying membrane structure, demonstrating how SIS can be combined with laser pyrolysis to create materials with enhanced properties.

## Results

### Characterization

Evidence for the successful incorporation of alumina into the PES membranes after SIS treatment can be seen in X-ray photoelectron spectroscopy (XPS) measurements (Fig. 1b), which show an absence of alumina before treatment and a strong intensity Al 2p peak after treatment consistent with Al_2_O_3_. Alumina incorporation was further confirmed by differences in membrane weight before (71.6 ± 0.1 g) and after (85.9 ± 0.5 g) SIS treatment, which suggests the treated films are 17% alumina by weight. The alumina content as determined by thermogravimetric analysis (TGA) was slightly higher at 28% (Supplementary Fig. 1a). This discrepancy could be explained by mass loss during the reaction of TMA with the PES membrane during SIS, making the actual loading higher than the mass change would suggest. Overall, however, the chemical composition of the original PES polymer is unchanged in the resulting organic–inorganic composite membrane, as confirmed by FTIR measurements (Supplementary Fig. 1b) and in agreement with the previous work^37^.

The LIG pyrolysis mechanism has been studied previously for other polymers, though not specifically for PES, and is thought to involve thermal decomposition of C–O and C–N bonds due to the rapid increase in temperature, followed by growth of ring clusters forming graphitic structures^38^. In the case of PES (and other sulfonated polymers), the transformation results in clusters with some insertion of sulfur in the graphene skeleton in the form of C-S-S and -C=S bonds^22^. Here, the conversion of PES to LIG is observed as a visual color change in the membranes from white to black (Supplementary Fig. 2) and is verified using Raman spectroscopy, which shows the presence of D, G and 2D bands characteristic of graphene containing materials (Fig. 1c). Optical images of the membranes lased at increasing laser powers (Supplementary Fig. 2) show that a critical laser power is required for conversion to LIG, as has been seen previously for other polymers^13^. The images also show that without the SIS treatment, the membranes transition through two regimes: at low but non-zero power, the membranes soften at their glass transition and become transparent (Supplementary Fig. 2); at a critical laser power, they exhibit the expected visual color change, though to not nearly as dark a color as the SIS-treated membranes and at higher laser powers than those required to convert SIS-treated membranes. SIS-treated membranes do not soften but instead graphitize directly, giving the first indication that the incorporation of alumina allows the membranes to resist changes to their nanoscale features during graphitization. Although the critical laser power that is required for LIG formation depends on lasing parameters such as the spacing between the laser scanning lines and laser speed, this same trend was observed at all setting tested, including the minimum spacing between scanning lines.

Cross-sectional scanning electron microscopy (SEM) images (Fig. 1d–f) demonstrate the powerful impact of SIS treatment on the membrane’s physical stability during lasing. Figure 1d shows the cross-sectional structure of a treated PES microfiltration membrane before lasing, along with a higher magnification image showing the micropores (Fig. 1g). When PES membranes without SIS treatment are lased, the membrane’s structure collapses into a dense bottom layer (Fig. 1f, k) and an exfoliated top layer (Fig. 1j). The membrane’s total cross-sectional thickness decreases to 38**–**58 μm compared to the initial 140 μm (Fig. 1d). In contrast, after lasing, SEM images of SIS-treated membranes have a thickness ranging from 90 to 132 μm (Fig. 1e, Supplementary Figs. 3–6) depending on the plane of the cross-section and the laser power used, showing that most of the membrane thickness is retained. Both the top 40.2 ± 1.0 μm of the membrane (high contrast in Fig. 1e, h), which is taken to be the lased region, and the remaining 85 ± 1.0 μm bottom layer (Fig. 1e, i) maintain an open and porous structure very similar to the starting membrane (see SI and Supplementary Fig. 7 for more detail about the membrane’s surface and cross-section structure). This indicates that graphitization happens in place without any macroscale deformation.

### Performance

**Fig. 2:**
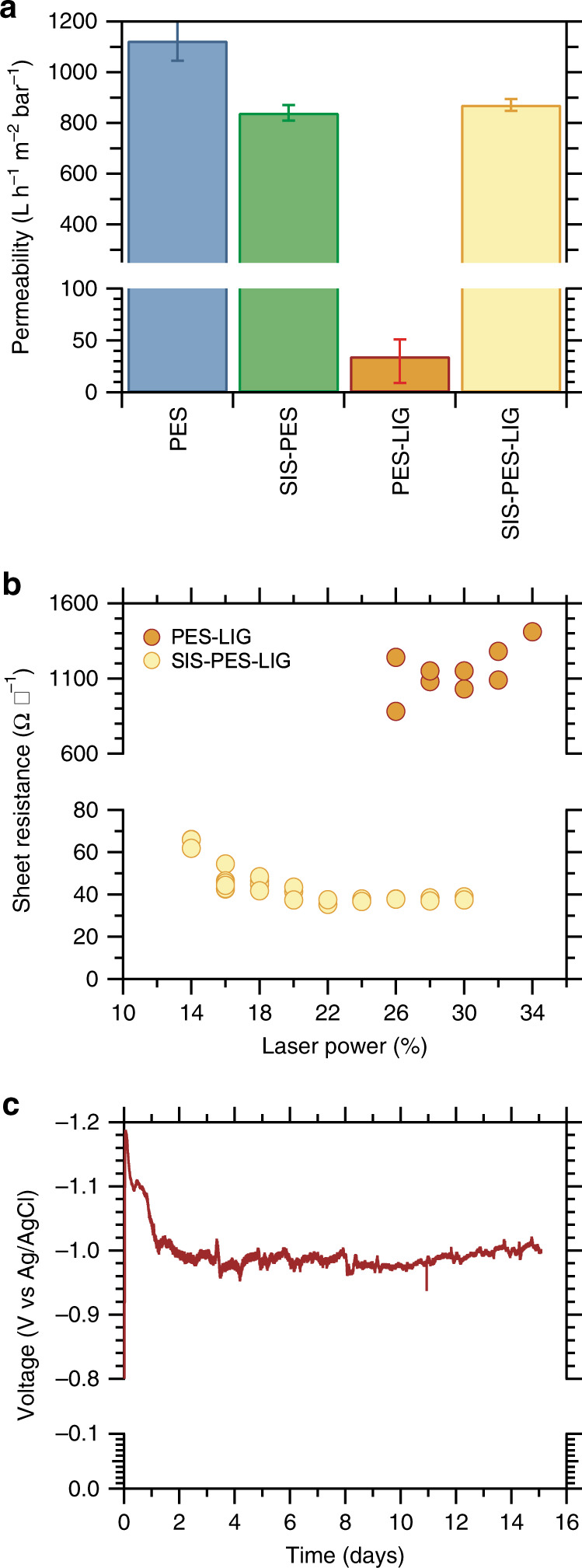
Performance of conductive membranes. **a** Permeability of PES membranes (with and without SIS treatment) before and after lasing. Error bars represent the range of data from repeated measurements. **b** Sheet resistance of lased membranes with and without SIS treatment as a function of the laser power used. **c** Applied potential required to maintain a reducing current of 10 mA cm^−2^ using SIS-PES-LIG electrodes over 14 days.

SIS-treated LIG membranes maintain the same permeability within uncertainty before and after lasing (872 Lm^−2^ h^−1^ bar^−1^) (Fig. 2a). Without SIS treatment, membranes show a dramatic decrease (from 1124 Lm^−2^ h^−1^ bar^−1^ to 35 Lm^−2^ h^−1^ bar^−1^) in permeability, owing to pore-closure in the subsurface.

In addition to maintaining their permeability, SIS-treated membranes also exhibit relatively low sheet resistance. Conductivity measurements of the LIG-coated membranes with and without SIS treatment (measured by the Van der Pauw method) as a function of laser power (Fig. 2b) show that the SIS-treated membranes achieve a sheet resistance of 37.7 ± 0.7 Ω □^−1^ or a conductivity of 660 S/m, based on the thickness of the conductive region seen in Fig. 1e. This sheet resistance is slightly higher than LIG formed from polyimide polymer, which show sheet resistance values as low as 15 Ω □^−1^, but is comparable to CNT membrane coatings which exhibit similar sheet resistances^3,39^. In contrast, the sheet resistance of lased membranes without SIS treatment (PES-LIG), which only graphitize and become conductive at higher laser powers (Supplementary Fig. 2), is an order of magnitude higher, in excess of 1000 Ω □^−1^. Their conductivity is also highly anisotropic: the two-point probe conductivity of these membranes is much higher along the direction of the laser path compared to perpendicular to the laser path.

Beyond high electrical conductivity, during operation, conductive membranes must be electrochemically stable enough to sustain either a capacitive voltage to electrostatically repel foulants or a faradaic current to electrochemically degrade foulants, strip scalants, and generate gas bubbles to remove accumulated contaminants. To verify their electrochemical stability, SIS-treated, lased membranes were subjected a reducing current of 10 mA/cm^2^ sufficient to perform water electrolysis^40^. Testing was performed on strips of the membrane surface that were dipped into 0.1 M NaCl electrolyte solution. To prevent contact between the electrolyte and the electrical contact wire due to wicking, only a small fraction of the lased area was dipped into the solution, far from the electrical contact point. In this configuration, membranes showed no loss in performance up to at least 14 days of continuous operation (Fig. 2c). An initial drop in the voltage required to drive 10 mA cm^−2^ of current density is attributed to the wicking of water further up the test strip during the first day of testing. Thus, laser-scribed SIS-treated membranes make excellent candidates for separations that require conductive membranes, or any technology where templated conductive structures are required.

### Mechanism of improved stability

**Fig. 3:**
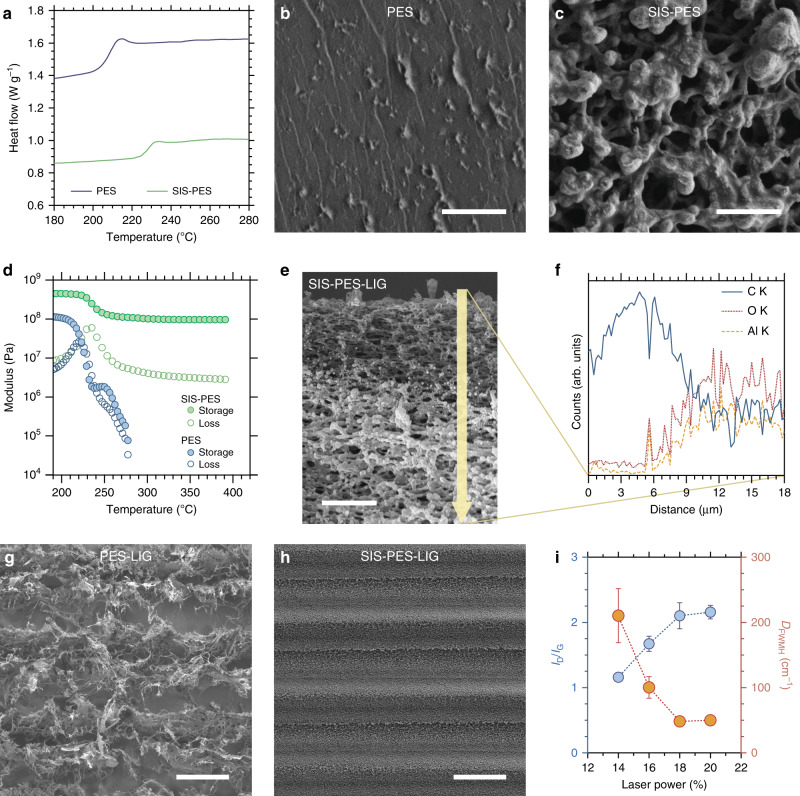
Mechanism of structural resilience during laser pyrolysis. **a** DSC scans of PES membranes with and without SIS treatment showing the similar glass transition temperatures of the polymer in the two membranes. SEM image of the **b** PES (scale bar 2 µm) and **c** SIS-treated PES membranes (scale bar 2 µm) after DSC measurement showing the different pore structure between the two membranes after heating. **d** Storage and loss modulus measurements of PES and SIS-treated PES membranes. **e** Cross-section SEM image of lased, SIS-treated membrane (scale bar 4 µm) and **f** its EDX line-scan along the yellow arrow shown in the SEM image. Lower magnification top surface SEM images of **g** lased PES (scale bar 100 µm) and **h** SIS-treated PES membranes (scale bar 100 µm). **i** D to G band intensity ratios and full width at half max of the D band of Raman spectra of SIS-treated PES membranes lased at increasing laser powers. Error bars represent the standard deviation of repeated measurements.

Given these favorable results, we explored the mechanisms behind the improved stability of SIS-treated membranes during lasing (i.e. suppression of deformation) and the improved conductivity of the LIG formed. One possible mechanism behind the structural stability during lasing is a change in the glass transition temperature (*T*_g_) of the membrane due to the addition of alumina. However, DSC analysis (Fig. 3a) shows only a minor increase in the glass transition temperature (*T*_g_) from 212 °C to 230 °C after SIS treatment. These values are consistent with previously reported *T*_g_ values for PES membranes^41,42^ and further indicate that the inclusion of alumina does not prevent the polymer in the membranes from undergoing a glass transition. SEM images of the membranes after the DSC measurements (i.e. after heating them above their glass transitions) (Fig. 3b, c) show that the PES completely loses its original porosity, while the alumina infiltrated sample remains mostly porous, with slight deformation. This suggests that, despite the similarity in *T*_g_ values, the presence of alumina alters the rheological properties of the SIS-PES membranes and stabilizes the membrane structure under elevated temperatures above the *T*_g_ of PES (the laser irradiation increases the PES temperature well above 230 °C)^13^. To test this hypothesis, we performed dynamic mechanical analysis (DMA) to measure the tensile storage (G′) and loss (G′′) modulus of PES and SIS-PES as a function of temperature (Fig. 3d). While the PES sample undergoes a full transition from a glassy state at 200 °C to terminal flow (i.e. liquid-like) behavior at 275 °C with only a mild entanglement plateau, SIS-PES shows a slight relaxation above 230 °C, but then exhibits a prolonged plateau in G′ (around 0.1 GPa) up to the instrumental limit of 400 °C. The solid-like properties of the SIS-PES sample are also reflected by the minimal sample elongation during testing, especially compared to PES, which yields over 100% and prematurely ends testing (Supplementary Fig. 8). The dramatic difference in mechanical properties between the two samples and the extended plateau in G′ of SIS-PES suggests that the alumina has formed a continuous network that stabilizes the original membrane structure well beyond the *T*_g_ of PES^43^.

While the infiltrated alumina is responsible for the mechanical structural resilience of the membrane during lasing, it is unlikely to be responsible for the improved conductivity and electrochemical stability of the LIG formed. Cross-sectional SEM and energy-dispersive X-ray spectroscopy (EDX) cross-sections of SIS-PES (Fig. 3e, f) show that after lasing, the top of the film is absent of aluminum introduced by SIS, likely due to sublimation under the high temperatures induced by the laser. At the LIG/PES interface, there is a small region where the alumina appears to have ripened into nanoparticles coating the polymer/LIG film but the conductive region of the resulting film is completely absent of the infiltrated alumina in its original crosslinked structure. Note that Fig. 3e is an SEM image of a membrane lased at lower laser power (14%) than the SEM image shown in Fig. 1e, which is why the thickness of the conductive layer is different. Further analysis of the PES-LIG and SIS-PES-LIG using XPS does not reveal significant chemical compositional differences. Both materials show primary C 1s peaks at a binding energy of 284.4 eV in XPS fine scans, consistent with sp2-bonded carbon^13^, with some additional higher binding energy peaks (Supplementary Fig. 9). While the PES-LIG shows a greater intensity of higher binding energy carbon peaks (286.2 eV), suggesting more ether carbon remains in the films after laser treatment, it is unlikely this difference in composition would lead to such a drastic difference in sheet resistance. Al 2p fine scans of the SIS-PES-LIG (Supplementary Fig. 10) also show that the aluminum within the film has remained in an oxide form, eliminating the possibility for the formation of Al metal. Raman spectra of PES-LIG and SIS-PES-LIG also do not exhibit significant enough differences to account for differences in conductivity. The D to G peak ratios in Raman spectra of carbonaceous materials are often used to make qualitative statements about the nature of LIG such as the crystallite size of graphitic clusters^44^. The peak ratios of the Raman spectra for PES-LIG and SIS-PES-LIG (Fig. 1c) suggest similar crystallite sizes ranging from 7–10 nm. However, LIG from untreated PES have broader peaks indicating increased disorder in these films relative to the SIS-treated samples^45^. In addition, some spots on the untreated PES films showed significant fluorescence (Supplementary Fig. 11a), indicating the presence of regions with minimal LIG coverage. Surface SEM images of the lased PES membrane without SIS show a heavily exfoliated structure with order-of-magnitude larger features (Supplementary Fig. 11a), and continuous regions of non-porous polymer underneath and in between, which is consistent with these Raman spectra. In contrast, spectra from SIS-treated PES after lasing showed consistent bands (Supplementary Fig. 11b) and SEM images of these membranes show a more homogeneous structure, similar to the pore-structure of the starting membrane.

The drastic differences in conductivity are therefore likely due to the less homogenous coverage of the lased surface by LIG. While membranes with and without SIS treatment show similar LIG thickness after lasing (Fig. 1e, f), lower magnification top-down SEM images of PES-LIG (Fig. 3g) reveal that after laser scribing, the laser creates linear trench structures of graphitized regions, separated by large gaps of non-porous polymer. These gaps between LIG structures also explain the high degree of anisotropy in conductivity mentioned earlier. A structural anisotropy is also visible in SIS-treated membranes (Fig. 3h). Previous work has shown that differences in lasing conditions can lead to vastly different LIG morphologies, in part due to differences in the resulting polymer temperature and anneal rate^14^. Here, regions outside of the direct laser path are still graphitized without any loss of porosity, but they likely do not reach the same annealing temperature and therefore exhibit different morphology.

Ultimately, the temperature that is achieved during laser irradiation plays a critical role in the resulting pyrolysis process and is affected by a combination of factors, such as the total absorption of IR light by the polymer and alumina (when present), the heat capacity of the membranes with and without alumina (which can be qualitatively deduced from the DSC data (Fig. 3a)), the structural changes due to polymer softening, the loss of porosity (without alumina), the rate of heat dissipation, and the total mass of polymer irradiated. While the relative impact of each of these factors was outside the scope of this work, it can be reasonably concluded that the presence of alumina increases the resulting membrane temperature during laser irradiation, since there is a reduction in critical laser power required for graphitization (Supplementary Fig. 2): the SIS-PES membranes can reach a higher temperature with lower laser energy and thus require less laser power to achieve the temperature required for graphitization.

The saturation in sheet resistance of the SIS-treated membranes above a laser power of 20–22% is also partially explained by the anisotropy in lased area. Initially, increasing the laser power is correlated with improved conductivity (Fig. 2b), as a greater fraction of the top surface of the membrane is converted to LIG and the laser continues to penetrate deeper into the membrane bulk. At 14% power, the laser is able to convert only parts of the membrane to LIG, which is seen as dark regions with low alumina content in surface SEM images, EDS maps, and EDS line scans (Supplementary Fig. 12). Cross-sectional images at 14% power show that the dark regions are part of a hemispherical path scribed by the laser (Supplementary Fig. 12). As the laser power is increased, the radius of this path grows and neighboring paths overlap until full surface coverage is achieved at which point the conductivity also saturates. We analyzed Raman spectra of the lased surfaces at different powers to see if higher laser powers changed the graphitic nature of the formed LIG. Between 14 and 18% laser power, there is an increase in the D to G peak intensity and area ratio, a narrowing of the D band full width at half max (Fig. 3i), and an emergence of a 2D peak (Supplementary Fig. 13). These changes plateau at higher powers, mirroring the conductivity measurements. These trends seen in the Raman spectra indicate that although initially, higher laser power leads to less disorder in the type of defects found on the LIG and increased stacking of graphitic clusters, defects found on the graphitic regions persist even at high powers^46^. Thus, continued improvement in the sheet resistance of the membranes will require alternative approaches, such as other chemical treatments besides alumina.

Interfaces and interfacial properties play a central role in many technologies other than membranes at the water and energy nexus^2^. PES membranes are taken as a prototypical example of porous polymers where nanosized features need to be preserved during laser scribing. The method of combining SIS of organometallic precursors into polymers with LIG formation described in this paper can be generalized to other applications where maintaining micro and nano-sized features of polymers at temperatures well above their *T*_g_ is desired. An even broader advantage of this approach is the improved mechanical and chemical properties that are observed at these high temperatures, without the need to change the chemistry of the underlying polymer^28^. Other modification approaches that could potentially be used, such as crosslinking of the polymer, would alter the polymer chemistry, potentially altering the material’s LIG forming properties. Since stability of LIG formed from polymers is a known concern^47^, the method described herein could be beneficial for all other polymers used for LIG formation as well.

In summary, we present a simple, solvent-free process for making conductive membrane coatings without altering underlying polymer structure. We demonstrate how infiltration with alumina stabilizes the PES membrane against deformation above the glass transition temperature^37^, allowing it to maintain its structure during laser treatment. These membranes are shown to be more conductive than LIG formed directly from the bare polymer, are electrochemically stable and maintain their permeability after lasing. These results demonstrate the immense versatility of hybrid polymer-ceramic materials as a promising class of materials to be used in conjunction with the LIG process.

## Methods

### Materials

Symmetric polyethersulfone (PES) membranes (200 nm pores, 47 mm diameter) were purchased from Pall Corporation (product number 60301) and used as received. Trimethylaluminum (TMA) was purchased from Strem Chemicals and used without further purification.

### SIS

Sequential infiltration synthesis was performed in the Center for Nanoscale Systems cleanroom using an Arradiance GEMStar-8 Bench-Top ALD system. Up to six PES membranes were placed on the reaction chamber stage along with a control silicon wafer to measure the nominal film thickness. Before depositions, samples were allowed to purge for a minimum of 20 min in 200 sccm of nitrogen and under vacuum at the reactor temperature of 100 °C. An alternating sequence of TMA and water was then dosed into the reaction chamber, where one set of TMA +Water pulse and purge steps is considered one “cycle” of the process. The mechanism for the TMA and water reaction is shown in Supplementary Fig. 13. In one reactant pulse, the nitrogen flow rate is reduced to 5 sccm with pumping turned off, allowing the reaction chamber to slowly fill with nitrogen. One of the two reactants is then pulsed into the chamber for 0.5 s before allowing the reactant and nitrogen to “soak” into the PES membranes for 5 min. Next, the pump valve was re-opened and the membranes were allowed to purge in 100 sccm of nitrogen for 10 min. Once all cycles were finished, the SIS-treated membranes were removed from the reactor while still hot. In this work, twelve of the above TMA+Water cycles were used in all presented data. More work will be needed to fully discern the relationship between the number of cycles, cycle times, and membrane properties.

### LIG formation

Laser treatment used to form LIG was performed on PES and SIS-PES membranes using a CO_2_ laser cutter system (VersaLASER, VLS 2.3, 10.6 μm, 25 W laser) at a speed of 50% (measured to be ~600 mm s^−1^), image density of 4, and laser power ranging from 10 to 30%. The laser focus was always set to 0.04 in above the membrane surface, to partially defocus the laser. The image density refers to the spacing between raster lines during lasing and can have a maximum value of 7 (higher number indicates closer spacing).

### Materials characterization

SEM images were obtained on a Hitachi Regulus 8100 SEM. XPS regional scans were collected on a Thermo Scientific K-Alpha system with an Al Kα X-ray source, with a spot size of 400 µm^2^. Fine scans were averaged over 10 scans (25 ms dwell time) with a pass energy of 50 eV and a 0.1 eV step size.

TGA and DSC analysis were done on a Q series TGA Q500 and DSC Q20 (TA instruments), respectively. For the DSC analysis, ~2.5 mg of PES or SIS-PES membrane was cut to fit into a hermetic aluminum pan and sealed. The temperature was ramped to 300 °C and back to −20 °C at a rate of 10 °C min^−1^ for 2 cycles.

Raman spectra were acquired using a Horiba LabRAM 800 HR spectrometer at 633 nm laser excitation. The laser was focused on the sample using a ×100 objective under reflected illumination and measurements were done with a 400 nm hole.

FTIR spectroscopy was performed using a Thermo Fisher FTIR6700 Fourier Transform Infrared Spectrometer using a germanium ATR plate and a mercury-cadmium-telluride detector chilled with liquid nitrogen. The uncoated ATR plate was used as a background and plotted data represents the average of 200 scans, with a step size of 4 cm^−1^.

Conductivity measurements were performed using an MMR Variable Temperature Hall Measurement System, operating at room temperature in the Van der Pauw configuration. 1 cm^2^ samples were used, with each measurement optimized for maximum allowable current.

Dynamic mechanical analysis (DMA) was conducted on a DMA Q 800 and analyzed with TA Universal Analysis software. Sample membranes were cut into rectangles with testing geometries of approximately 10 × 8 × 0.125 mm and equilibrated at 190 °C for 5 min before testing. Temperature ramps were collected at a frequency of 1 s^−1^ with a strain amplitude of 0.05% strain and a ramp rate of 3 °C min^−1^.

For permeability measurements, 5 mm diameter circular holes were laser cut on PET masks and membranes were epoxied onto the masks so that an area of 19.6 mm^2^ was exposed for water permeation. The attached membrane (along with the PET mask) was then placed in a vacuum filtration glassware with 25 mm filter glass support. Permeability was measured in the vacuum filtration setup (Gast pump with max pressure of 60 psi) by monitoring the decrease in feed water volume from a video recording. The use of PET masks and vacuum filtration setup was chosen due to a lack of resilience in larger area SIS-PES-LIG membranes to pinching from o-rings in a standard dead-end filtration cells. However, anecdotal evidence suggests that membranes can be made more resilient to pinching by modifying SIS conditions and future work in this area is needed.

Electrochemical stability tests were performed using Bio-logic SP-300 potentiostat in a three-electrode cell setup, using a platinum wire as the counter electrode and a Ag/AgCl reference electrode purchased from Fischer Scientific (Fisherbrand accumet, glass body). A 0.1 M NaCl solution was used as the electrolyte. Electrochemical impedance spectroscopy was performed to measure the series resistance of the cell (~100 Ω) before running chronopotentiometry at a reducing potential of 10 mA cm^−2^ for 14 days. The measured potential was corrected for the series resistance (iR). The Data presented are representative.

## Supplementary information

Supplementary Information

Peer Review File

## Data Availability

The data that support the findings of this study are available from the corresponding author upon reasonable request.
