# Supplementary material for: Preserving nanoscale features in polymers during laser induced graphene formation using sequential infiltration synthesis
Source: Nat Commun. 2020 Jul 20;11:3636. doi: 10.1038/s41467-020-17259-5 (PMC7371709; doi:10.1038/s41467-020-17259-5)
Supplement: Supplementary file 1 — Supplementary Information [file 41467_2020_17259_MOESM1_ESM.pdf]

Supplementary Information

Preserving Nanoscale Features in Polymers During Laser-Induced Graphene Formation Using Sequential Infiltration Synthesis

David S. Bergsman[1,‡], Bezawit A. Getachew[1,‡], Christopher B. Cooper[2], Jeffrey C. Grossman[1]

[1]Department of Materials Science and Engineering, Massachusetts Institute of Technology, MA, USA.

[2]Department of Chemical Engineering, Stanford University, Stanford, CA, USA.

[‡]These authors contributed equally to this work.

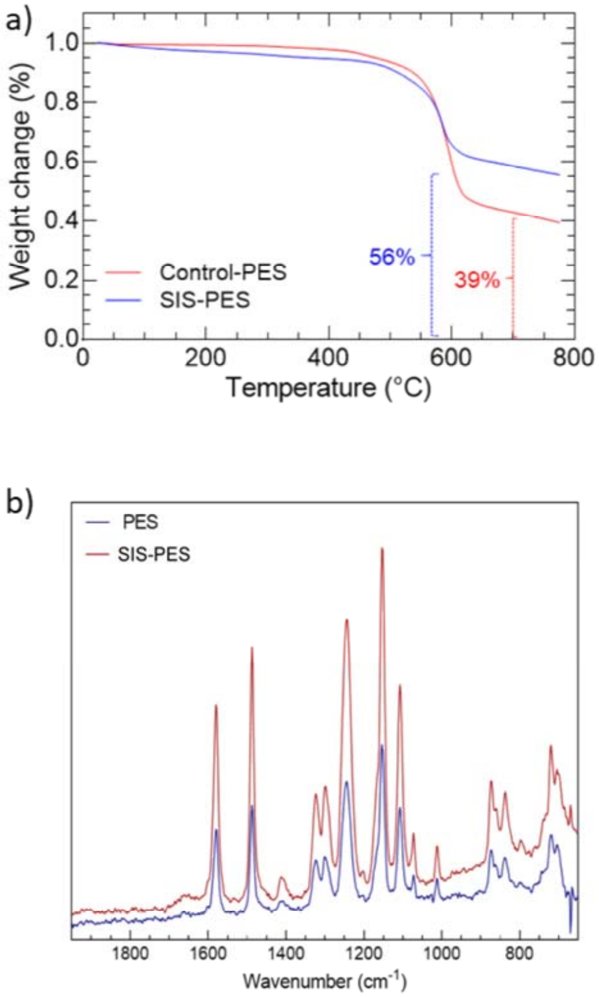

Supplementary Figure 1. (a) TGA analysis of control-PES and SIS-PES membranes. The incorporation of alumina in the SIS-PES membranes leads to a higher percentage of the membrane remaining after annealing. (b) FTIR of PES membranes before and after SIS treatment

Supplementary Note 1

The content of alumina in the SIS-PES samples was calculated using the following two supplementary equations, where P is the polymer content of the membrane and A is the alumina content of the membrane. Before performing TGA, the fraction of polymer and alumina must total 1:

$$P + A = 1 \tag{1}$$

After performing TGA, the remaining polymer content was 0.39% without alumina present. Assuming that the alumina remains unchanged during TGA (it remains stable up to 780 °C) and assuming that the presence of alumina does not change the annealing of the polymer, then after performing TGA, the fraction of polymer and alumina remaining in the film is:

$$0.39P + A = 0.56 \tag{2}$$

Solving, we see that the alumina must total 28% of the infiltrated polymer.

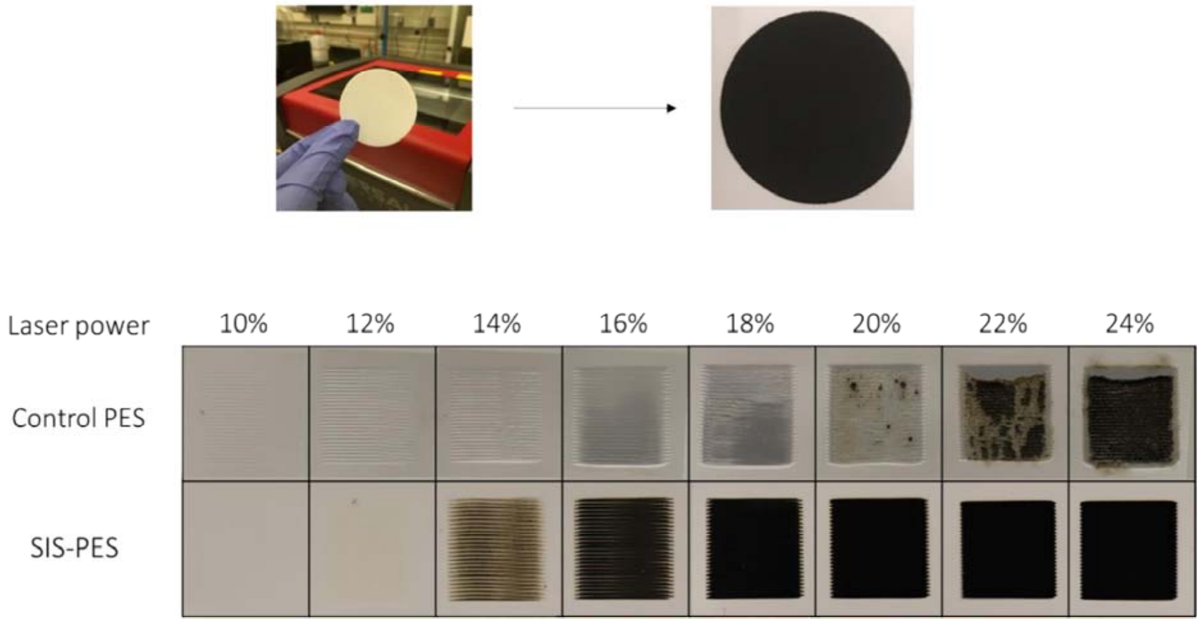

Supplementary Figure 2: Optical image of a PES membrane coupon before and after lasing (top) and of lased PES rectangles (with and without SIS) at different laser powers

Supplementary Note 2: Surface structure of lased membranes

The laser creates a hemispherical pattern where regions along the direct path of the laser (i.e. the middle of the raster line) are graphitized more than the edge of the laser path. This effect is seen in the surface SEM images (Fig. S3) where the dark regions are fully graphitized LIG regions and the lighter bands are regions that were not in the direct path of the laser. The width of the dark and light bands that is created depends on the laser power used as shown Figure S3. The membranes then have a corrugated surface structure such as the one shown in Figure S4.

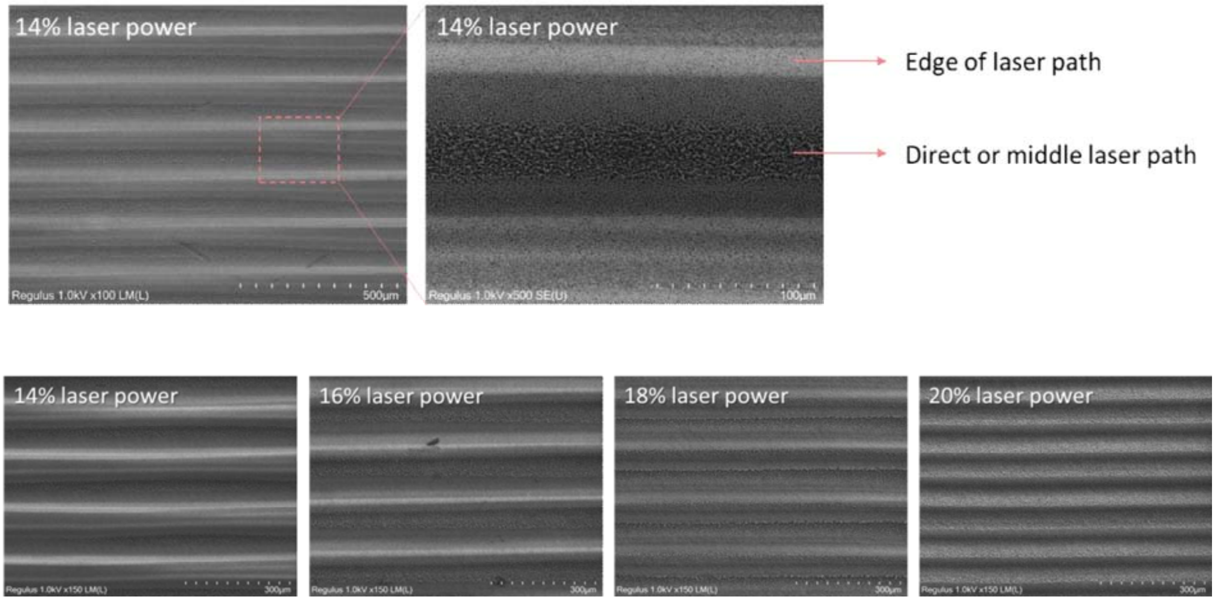

Supplementary Figure 3. Surface SEM images of lased membranes showing the difference between the middle and edge of the laser path, as well as the effect of increasing laser power.

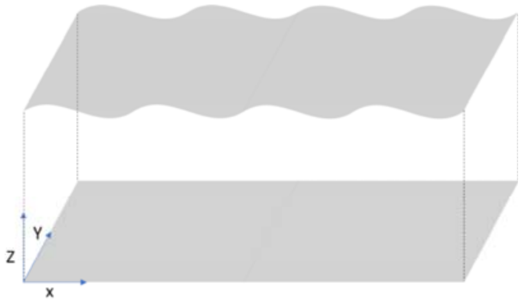

Supplementary Figure 4. Corrugated surface structure of membranes after lasing

This surface structure means that the membrane’s cross-section SEM images are heavily dependent on the plane of the cross section. When the membrane is cut along the xz plane, for example, the top of the cross-section will be a wavy pattern, such as the SEM image shown in Fig. S5a. Figure S5b is the same SEM image as Fig. S5a taken with the upper SEM detector which is more prone to charging and therefore gives an indication of the more conductive region of the cross-section with LIG and the unaltered PES layer which is insulating.

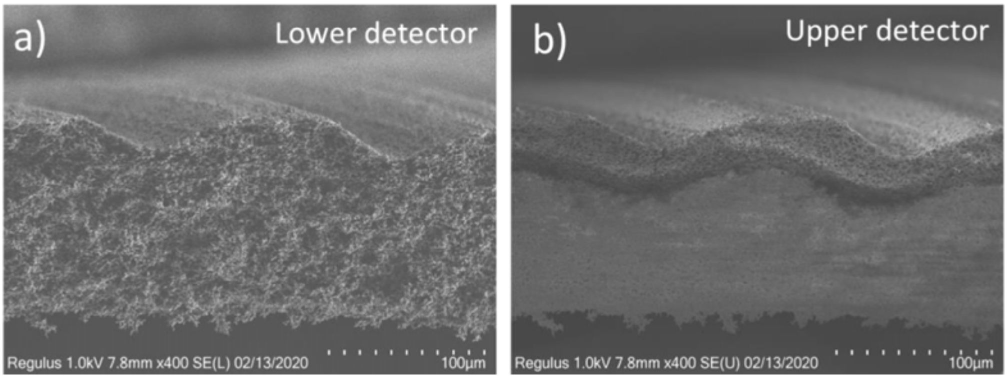

Supplementary Figure 5. Cross-section SEM image of a lased membrane using the lower (a) and upper (b) detectors of the microscope.

If the membrane is cut along the yz plane, then the top will have a linear structure such as the one shown in Fig. S11b. When the membrane is cut along the yz plane, the SEM image captured will also depend on whether the membrane is cut along the plane of a ridge or a groove. When the membrane is cut along the plane of a groove, the cross-section image will also include the ridge—although the ridge will be out of focus. This is what is responsible for the out of focus part of the image on Fig. 1e and Fig. S11b. As shown Figure S11, the thickness of the membrane can be verified by the consistency of the thickness measured from different cross section planes.

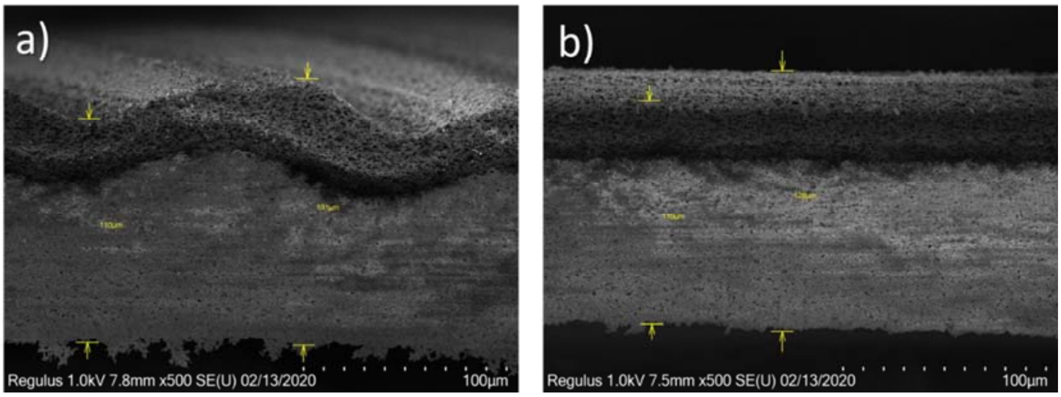

Supplementary Figure 6. Cross section SEM images of a lased membrane fractured along different planes.

Higher resolution images of the top two bright layers of Figure 1e (reproduced below, along with the same image taken with the upper detector) show that the top most layer is made up of longer and thinner LIG strands, consistent with what is expected from the middle, more graphitized region of the membrane. The second bright layer on the other hand has shorter and thicker strands which is consistent with the edge of the path of the laser. The rest of the higher resolution images along the cross section show the original PES porous structure.

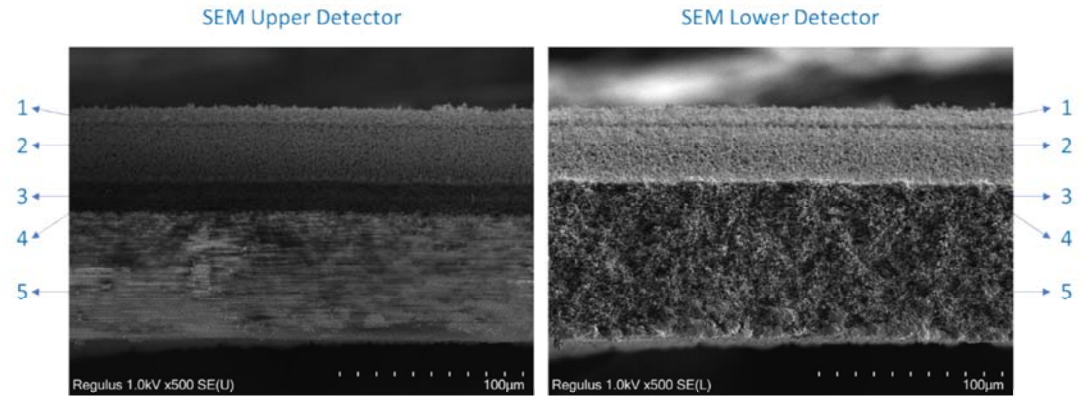

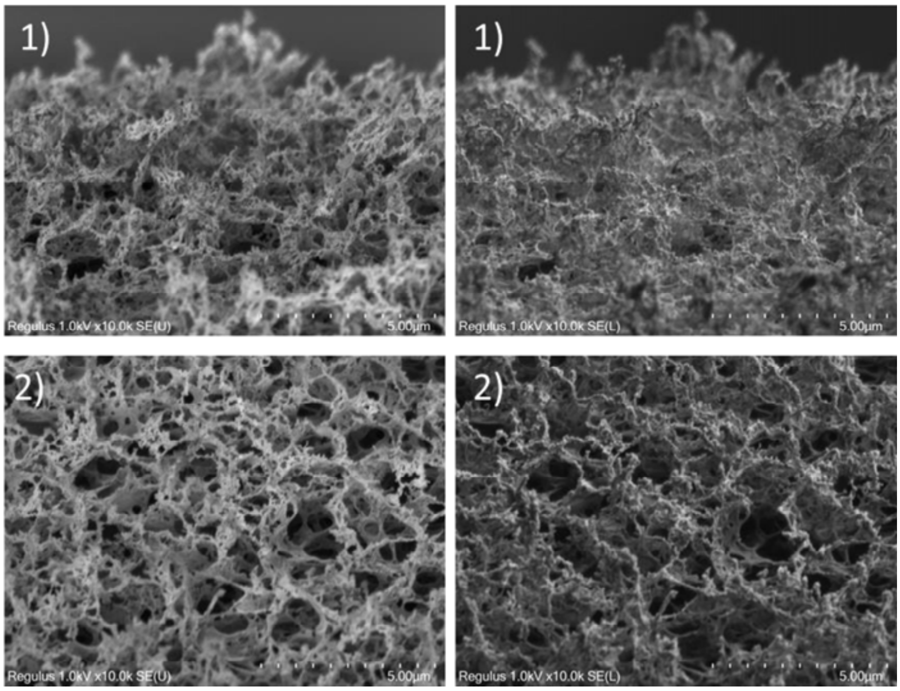

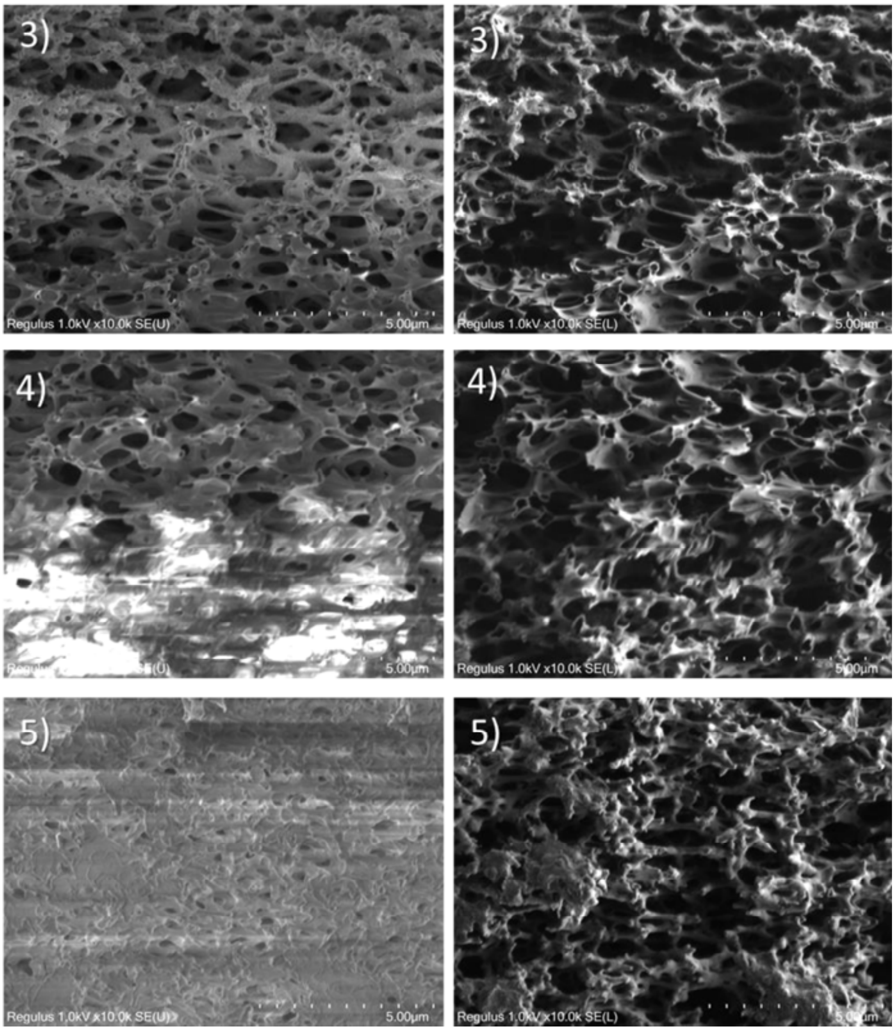

Supplementary Figure 7. SEM images of lased membranes showing the difference in structure along the cross-section of the membrane.

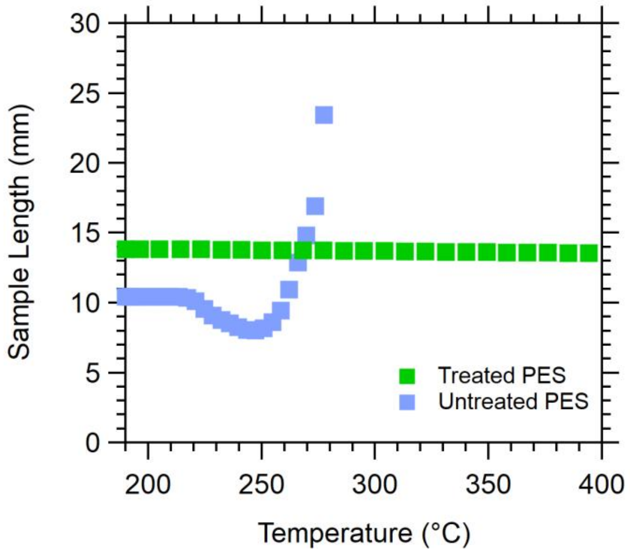

Supplementary Figure 8: Sample elongation measurements at increasing temperatures during dynamic mechanical analysis

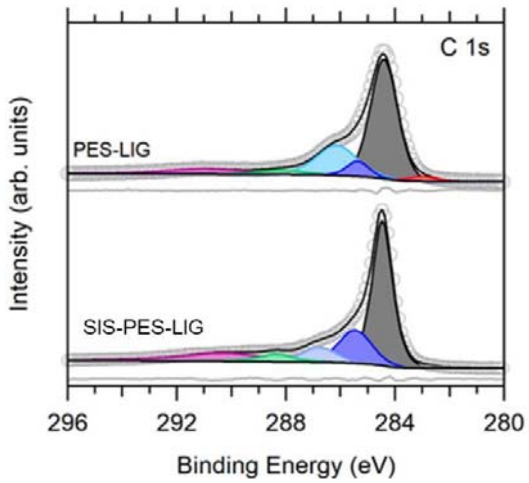

Supplementary Figure 9. XPS regional scans of carbon 1s for PES-LIG and SIS-PES-LIG

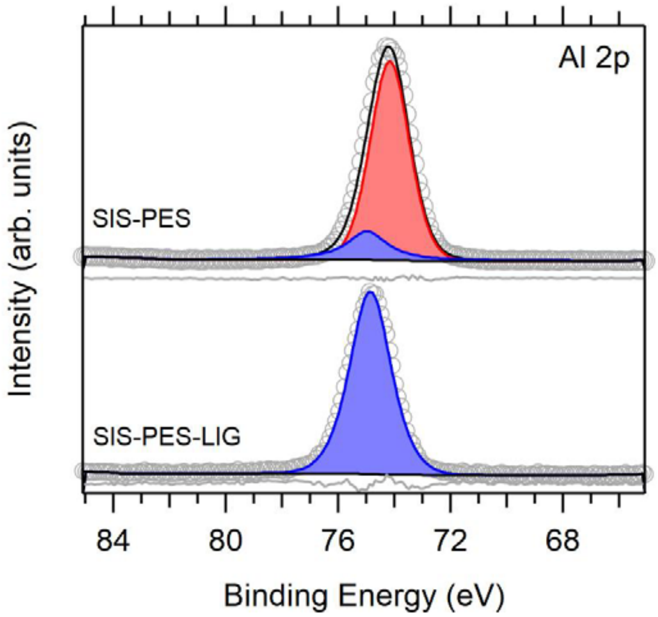

Supplementary Figure 10. XPS regional scans of aluminum 1s for SIS-PES and SIS-PES-LIG.

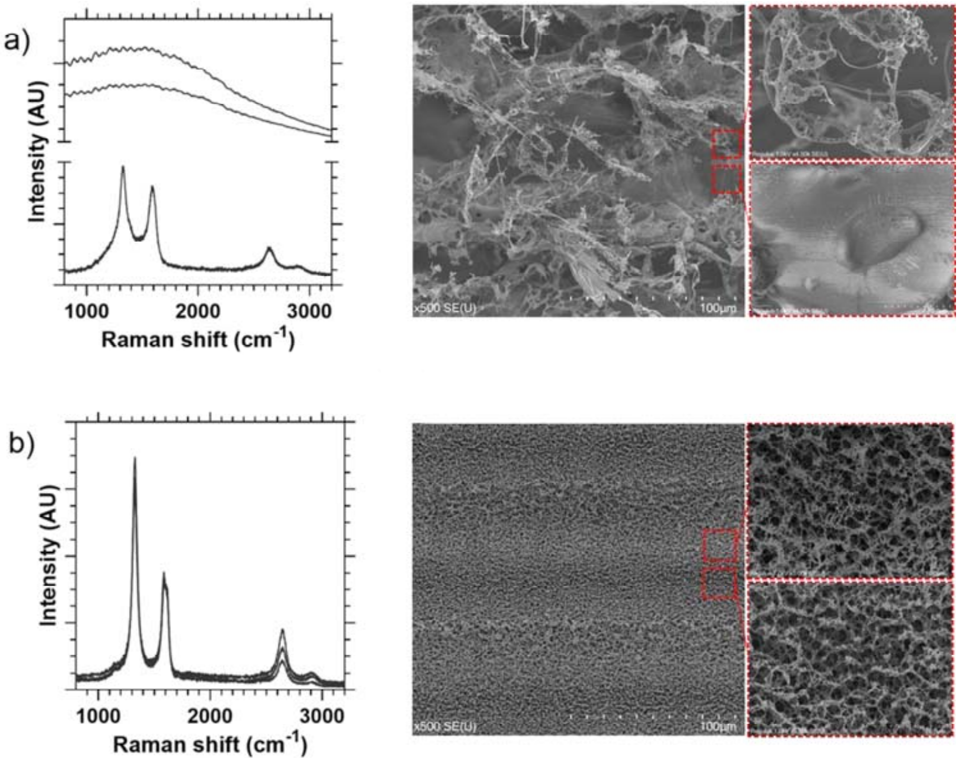

Supplementary Figure 11. Raman spectra and surface SEM images of (a) untreated and (b) SIS treated PES after lasing from three different spots. Two of the spots on the untreated PES show significant fluorescence without any discernible peaks while one spot has spectra with clear D, G and 2D bands.

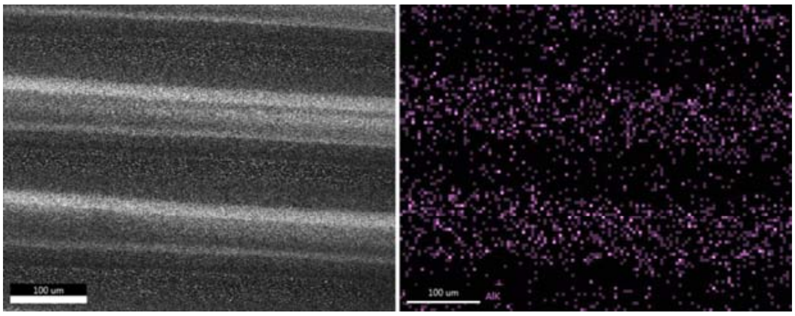

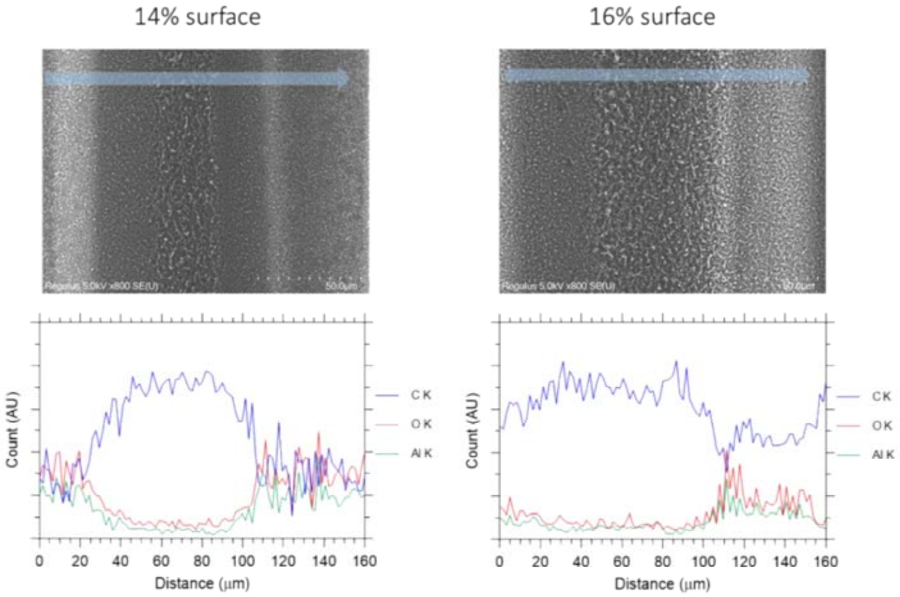

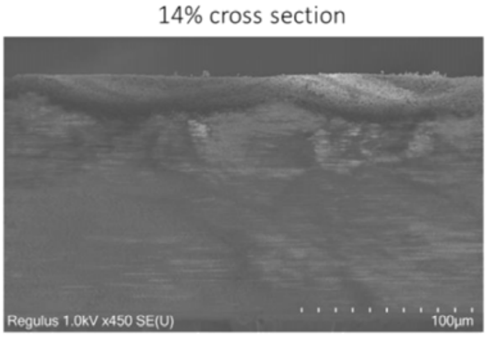

Supplementary Figure 11. Surface SEM images, EDX map, and EDX line scans of lased SIS-PES membranes (top). SEM cross-section images of a membranes lased at 14% power

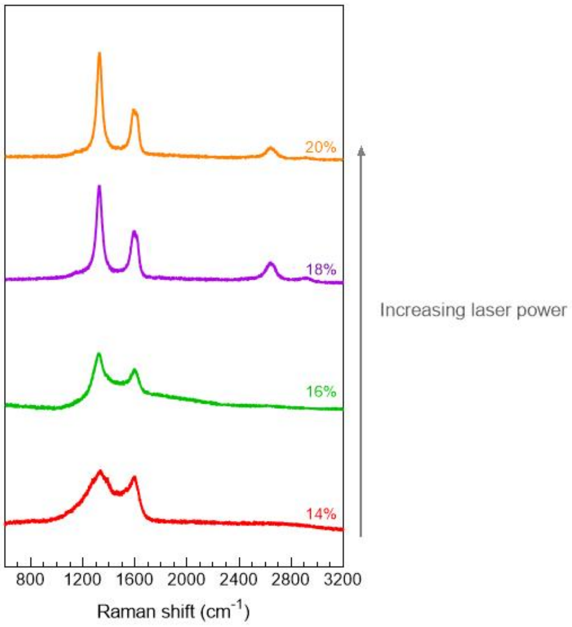

Supplementary Figure 12. Raman spectra of SIS treated PES at different laser powers.

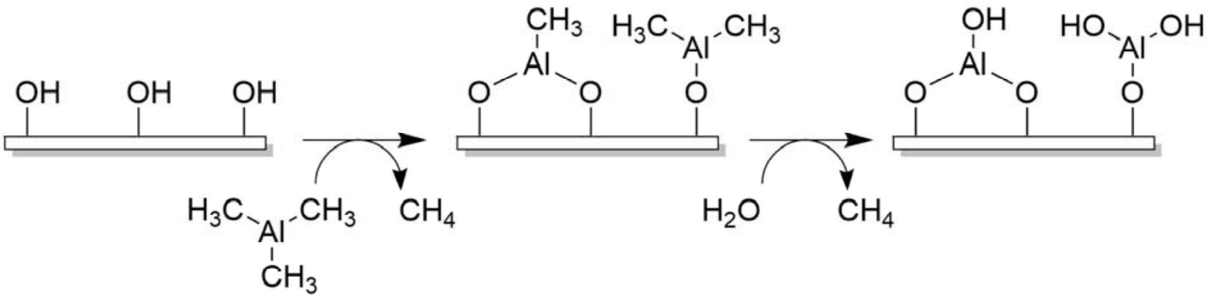

Supplementary Figure 13. Reaction mechanism for the TMA + water reaction.
