# Supplementary material for: Preserving nanoscale features in polymers during laser induced graphene formation using sequential infiltration synthesis
Source: Nat Commun. 2020 Jul 20;11:3636. doi: 10.1038/s41467-020-17259-5 (PMC7371709; doi:10.1038/s41467-020-17259-5)
Supplement: Supplementary file 2 — Peer Review File [file 41467_2020_17259_MOESM2_ESM.pdf]

Editorial Note: This manuscript has been previously reviewed at another journal that is not operating a transparent peer review scheme. This document only contains reviewer comments and rebuttal letters for versions considered at Nature Communications.

REVIEWERS' COMMENTS:

Reviewer #1 (Remarks to the Author):

The authors have made substantive improvements to this manuscript in response to the original referee reports. Specifically, they have added several pieces of additional data (e.g. TGA and new EM) and improved the referencing and context for non-specialists. This work is now suitable for publication.

Reviewer #2 (Remarks to the Author):

After carefully reading the authors' responses, I found the authors have addressed my questions and concerns in the revised version of the manuscript. Therefore, I recommend the publication in its current form.

Reviewer #3 (Remarks to the Author):

After reading through the manuscript, the reviewers addressed all concerns raised in my former review-- I thank the authors for well-addressing my concerns. Also the impact factor of Nature Communications seems fitting for this work. I therefore suggest to accept without further revisions.
